# Comparison of synthesized and acquired high *b*-value diffusion-weighted MRI for detection of prostate cancer

**DOI:** 10.1186/s40644-024-00723-6

**Published:** 2024-07-08

**Authors:** Karoline Kallis, Christopher C. Conlin, Allison Y. Zhong, Troy S. Hussain, Aritrick Chatterjee, Gregory S. Karczmar, Rebecca Rakow-Penner, Anders M. Dale, Tyler M. Seibert

**Affiliations:** 1 Department of Radiology, University of California San Diego Health, La Jolla, San Diego, CA USA; 2 Department of Radiation Medicine and Applied Sciences, University of California San Diego Health, La Jolla, CA USA; 3 Department of Neurosciences, University of California San Diego Health, La Jolla, San Diego, CA USA; 4 Halıcıoğlu Data Science Institute, University of California San Diego, La Jolla, San Diego, CA USA; 5 Department of Bioengineering, University of California San Diego Jacobs School of Engineering, La Jolla, San Diego, CA USA; 6 Department of Radiology, University of Chicago, Chicago, IL USA; 7 Sanford J. Grossmann Center of Excellence in Prostate Imaging and Image Guided Therapy, University of Chicago, Chicago, IL USA

**Keywords:** Diffusion-weighted imaging, Prostate cancer, Synthetic high *b*-values, Restricted Spectrum Imaging

## Abstract

**Background:**

High *b*-value diffusion-weighted images (DWI) are used for detection of clinically significant prostate cancer (csPCa). This study qualitatively and quantitatively compares synthesized DWI (sDWI) to acquired (aDWI) for detection of csPCa.

**Methods:**

One hundred fifty-one consecutive patients who underwent prostate MRI and biopsy were included in the study. Axial DWI with *b* = 0, 500, 1000, and 2000 s/mm^2^ using a 3T clinical scanner using a 32-channel phased-array body coil were acquired. We retrospectively synthesized DWI for *b* = 2000 s/mm^2^ via extrapolation based on mono-exponential decay, using *b* = 0 and *b* = 500 s/mm^2^ (sDWI_500_) and *b* = 0, *b* = 500 s/mm^2^, and *b* = 1000 s/mm^2^ (sDWI_1000_). Differences in signal intensity between sDWI and aDWI were evaluated within different regions of interest (prostate alone, prostate plus 5 mm, 30 mm and 70 mm margin and full field of view). The maximum DWI value within each ROI was evaluated for prediction of csPCa. Classification accuracy was compared to Restriction Spectrum Imaging restriction score (RSIrs), a previously validated biomarker based on multi-exponential DWI. Discrimination of csPCa was evaluated via area under the receiver operating characteristic curve (AUC).

**Results:**

Within the prostate, mean ± standard deviation of percent mean differences between sDWI and aDWI signal were -46 ± 35% for sDWI_1000_ and -67 ± 24% for sDWI_500_. AUC for aDWI, sDWI_500,_ sDWI_1000_, and RSIrs within the prostate 0.62[95% confidence interval: 0.53, 0.71], 0.63[0.54, 0.72], 0.65[0.56, 0.73] and 0.78[0.71, 0.86], respectively.

**Conclusion:**

sDWI is qualitatively comparable to aDWI within the prostate. However, hyperintense artifacts are introduced with sDWI in the surrounding pelvic tissue that interfere with quantitative cancer detection and might mask metastases. In the prostate, RSIrs yields superior quantitative csPCa detection than sDWI or aDWI.

## Background

Diffusion-weighted imaging (DWI) is a critical component of multiparametric MRI for the detection and characterization of clinically significant prostate cancer (csPCa) [1]. The degree of diffusion-weighting in DWI is indicated by the *b*-value, with higher *b*-values corresponding to images with less signal where water in tissues diffuses more rapidly [2]. High *b*-values are used for their greater tumor conspicuity and detection of even small lesions [3]. The Prostate Imaging – Reporting and Data System (PI-RADS v2.1) recommends the acquisition of high *b*-values (1400–2000s/mm^2^) for lesion detection, without precisely defining an optimal value for csPCa [4]. While clinically valuable, high *b*-values require more scan time and suffer from low signal-to-noise ratio (SNR) and increased susceptibility to artifacts due to microscopic motion or small fluctuations in local magnetic field. One common solution, permitted by PI-RADS, is to synthesize high *b*-value images by extrapolating signal from acquired low *b*-value images using a mono-exponential model [1, 5]. However, mono-exponential models do not adequately represent restricted diffusion in complex tissues [6, 7], possibly calling into question the accuracy of synthesized images.

More advanced DWI models have been developed to better account for tissue microstructure, including intravoxel incoherent motion imaging [8, 9], diffusion kurtosis imaging [10, 11], Vascular, Extracellular, and Restricted Diffusion for Cytometry in Tumor (VERDICT) [12–14], hybrid multidimensional MRI (HM-MRI) [15–18], and Restriction Spectrum imaging (RSI) [12, 19]. In RSI, the diffusion signal is modeled as a weighted sum of different compartments representing different tissue types [19, 20]. The RSI restriction score (RSIrs) is based on the model coefficient for the most restricted diffusion compartment and has been shown to be a useful biomarker for the detection of csPCa [20–22].

Studies have yielded contradicting results on whether synthesized *b*-values are clinically interchangeable with acquired DWI (aDWI) images. Liu et al*.* [23] compared various models, including the standard mono-exponential, for the detection of csPCa and concluded that non-linear fitting with various *b*-values is superior to simpler models. In contrast, other studies reported better image quality for synthetic DWI (sDWI) with a similar tumor detection rate in comparison to acquired DWI [5, 24–27].

In this study, we qualitatively and quantitatively analyzed the differences between acquired and synthesized high* b*-value images for detection of csPCa. Further, we evaluated acquired and synthesized high *b-*value DWI for detection of csPCa at the patient level. For comparison, we also evaluated RSIrs, a quantitative biomarker based on multi-compartment DWI that is known to perform well for patient-level csPCa detection.

## Methods

### Patient cohort

This retrospective study was approved by the institutional review board at UC San Diego (IRB 805394). The research was performed in accordance with the Declaration of Helsinki, and all relevant regulations. A waiver of consent was approved by the institutional review board for this study as there was minimal risk of harm to patients. The retrospective dataset was described previously [22]. Briefly, 440 consecutive men who underwent prostate MRI examination with a multi-*b*-value diffusion acquisition (compatible with Restriction Spectrum Imaging, RSI) between November 2017 and December 2020 were considered for inclusion. Patients were excluded if they had undergone prior treatment for prostate cancer or if there was no available biopsy result performed within 180 days of MRI acquisition. In total 151 patients were included in the study. Patient characteristics are summarized in Table 1.

**Table 1:** Patient Characteristics range *Q*_*1*_*-Q*_*3*_ Range between lower first quartile to upper third quartile, *csPCa* Clinically significant prostate cancer. *MRI* Magnet resonance imaging, *PSA* Prostate-specific antigen

Parameter	Specification	Value
Number of patients	Total	151
Age [a]	Median (range Q_1_-Q_3_)	66 (59–72)
Time from MRI to biopsy [d]	Median (range Q_1_-Q_3_)	16 (1–35)
PSA at time of MRI [ng/ml]	Median (range Q_1_-Q_3_)	7.3 (5.3–10.4)
Prostate volume [ml]	Median (range Q_1_-Q_3_)	45 (34–61)
PSA density [ng/ml^2^]	Median (range Q_1_-Q_3_)	0.16 (0.11–0.25)
Best available pathology	Systematic Targeted Systematic and Targeted Prostatectomy	7 17 85 42
PI-RADS Score (csPCa)	I II III IV V	0 5 (3) 27 (4) 55 (25) 64 (54)
Gleason Grade Group	Benign 1 2 3 4 5	25 40 38 20 16 12
Clinical Tumor stage	Negative Biopsy T1c T2a T2b T2c	25 94 13 11 8

MRI examinations were interpreted per routine clinical practice by ten board-certified (median of four years of experience) and subspecialty fellowship-trained radiologists. For all patients, suspicious lesions were contoured per PI-RADS v2.1 using MIM software (MIM Software, Inc; Cleveland, OH). For the present study, whole-gland prostate segmentation was performed using OnQ Prostate software (Cortechs Labs, San Diego, CA, USA). Clinically significant prostate cancer (csPCa) was defined as grade group ≥ 2. In patients who underwent prostatectomy, grade group was determined per final pathology report. Biopsy (typically systematic and targeted) and prostatectomy were performed according to clinical routine, and both were examined by board-certified pathologists. 86 of the 151 patients were found to have csPCa, while 65 had only benign tissue or grade group 1 cancer (further details in Table 1).

### MRI acquisition

All MRI acquisitions were performed on a 3T clinical GE scanner (Discovery MR750, GE Healthcare, Waukesha, WI, USA) using a 32-channel phased-array body coil surrounding the pelvis. Acquisition parameters are summarized in Table 2. A single axial DWI volume was acquired for each patient. *T*_*2*_-weighted reference images were acquired for all patients with field of view (FOV) identical to the DWI volume. RSI calculations were performed as described in prior studies [20–22].

**Table 2:** Acquisition parameters for clinical multi-parametric MRI; *DWI* = Diffusion-weighted imaging, *T2W* = T_2_ weighted MRI

Series	DWI	T2W
FOV [mm*mm]	240*120	320*320
Matrix (resampled dimensions)	96*48 (128*64)	320*320 (512*512)
Number of Slices	16	32
Slice thickness [mm]	6	3
TR [ms]	4500	6080
TE [ms]	68	102
*b*-values [s/mm^2^] (number of samples)	0 (2), 500 (6), 1000 (6), 2000 (12)	N/A

Post-processing of the image data was performed using in-house software in MATLAB (version R2017a, MathWorks, Natick, MA, USA). DWI images were corrected for *B*_*0*_ inhomogeneity distortions, gradient nonlinearity, and eddy currents [28–30]. Multiple acquired DWI samples at specific *b*-values were averaged together and normalized by median signal intensity of urine in the bladder at *b* = 0 s/mm^2^.

### Synthetic *b*-value computation

Synthetic high *b*-value DWI (sDWI) was calculated using the conventional, mono-exponential formula (see below) and using *b*-values up to 500 s/mm^2^ (sDWI_500_) or *b*-values up to 1000 s/mm^2^ (sDWI_1000_).

$$S\left(b\right)=S_0e^{-b\;ADC}$$

*S(b)* is DWI signal for a given *b*-value, *b*. *S*_*0*_ is the signal with no diffusion weighting. *ADC* is the apparent diffusion coefficient. sDWI was calculated for *b* = 2000s/mm^2^ to match the acquired high *b*-value DWI (aDWI). To explore the application of sDWI and aDWI for detection of significant cancer lesions outside of the prostate, sDWI and RSIrs were additionally calculated for one representative patient with csPCa and bone metastasis.

### Data analysis

All data analysis was performed using in-house MATLAB scripts (version R2021a, MathWorks, Natick, MA, USA). Quantitative differences between sDWI and aDWI were estimated by a voxel-wise comparison of the images. Relative deviations were calculated for three different regions of interest (ROIs): prostate, prostate plus a margin of 5 mm, and the whole field of view (FOV) using the following formula:

$$\Delta S=\frac{1}{N}\sum_{i=0}^{N}\frac{{S}_{s}-{S}_{a}}{{S}_{a}}$$

where S_s_ is the synthetic signal intensity, S_a_ the acquired signal and N the number of voxels in the considered images. Mean and standard deviation of ΔS over all patients are reported. A negative value indicates that the acquired signal intensity is higher than the synthesized signal intensity. Further, violin plots were generated for the 50^th^, 95^th^, and 98^th^ percentile of signal intensity within several ROIs: prostate; prostate plus margin (5 mm, 30 mm, or 70 mm); and the whole FOV. For the whole FOV, values higher than 3000 signal intensity units (SIU) were capped and set to 3000 SIU. Violin plots present the median value in combination with the kernel density distribution [31].

Lesion conspicuity was evaluated using the contrast-to-noise ratio (CNR) between lesion and surrounding prostate tissue. CNR is defined as the following:

$$CNR=\frac{({\mu }_{lesion}-{\mu }_{prostate})}{\sqrt{{{\sigma }_{lesion}}^{2}+{{\sigma }_{prostate}}^{2}}}$$

where μ is the mean signal of the ROI under consideration and σ the standard deviation. CNR was evaluated for all patients and patients diagnosed with csPCa. A higher CNR indicates a better tumor conspicuity [32]. Significant differences between CNRs of different images and patient cohorts were tested using two sample t-test with a confidence level of 0.01.

Prediction of whether csPCa was found on biopsy was also evaluated for aDWI, sDWI, and RSIrs. RSIrs is a quantitative cancer biomarker based on a multi-exponential DWI model (four compartments) and has been previously shown to be more accurate than conventional DWI [20–22]. Computation of RSIrs for this dataset was performed previously and is described in detail in previous publications [20–22]. Briefly, the coefficient for the slowest diffusion compartment (corresponding to intracellular restricted diffusion) was normalized by the median signal within the prostate on *b* = 0 s/mm^2^ images. The maximum aDWI, sDWI, or RSIrs value within each considered ROI was used as the predictor variable [22]. This is analogous to the maximum standard uptake value (SUV) in quantitative Positron Emission Tomography (PET) imaging. Receiver-operating characteristic (ROC) curves were calculated, and the area under the curve (AUC) reported for aDWI, sDWI, and RSIrs. The false positive rate at 90% sensitivity (FPR90) was also reported for each metric to illustrate performance at one threshold [21]. AUC and FPR90 were compared using bootstrap (*N* = 10,000) 95% confidence intervals and *p*-values.

## Results

**Fig. 1:**
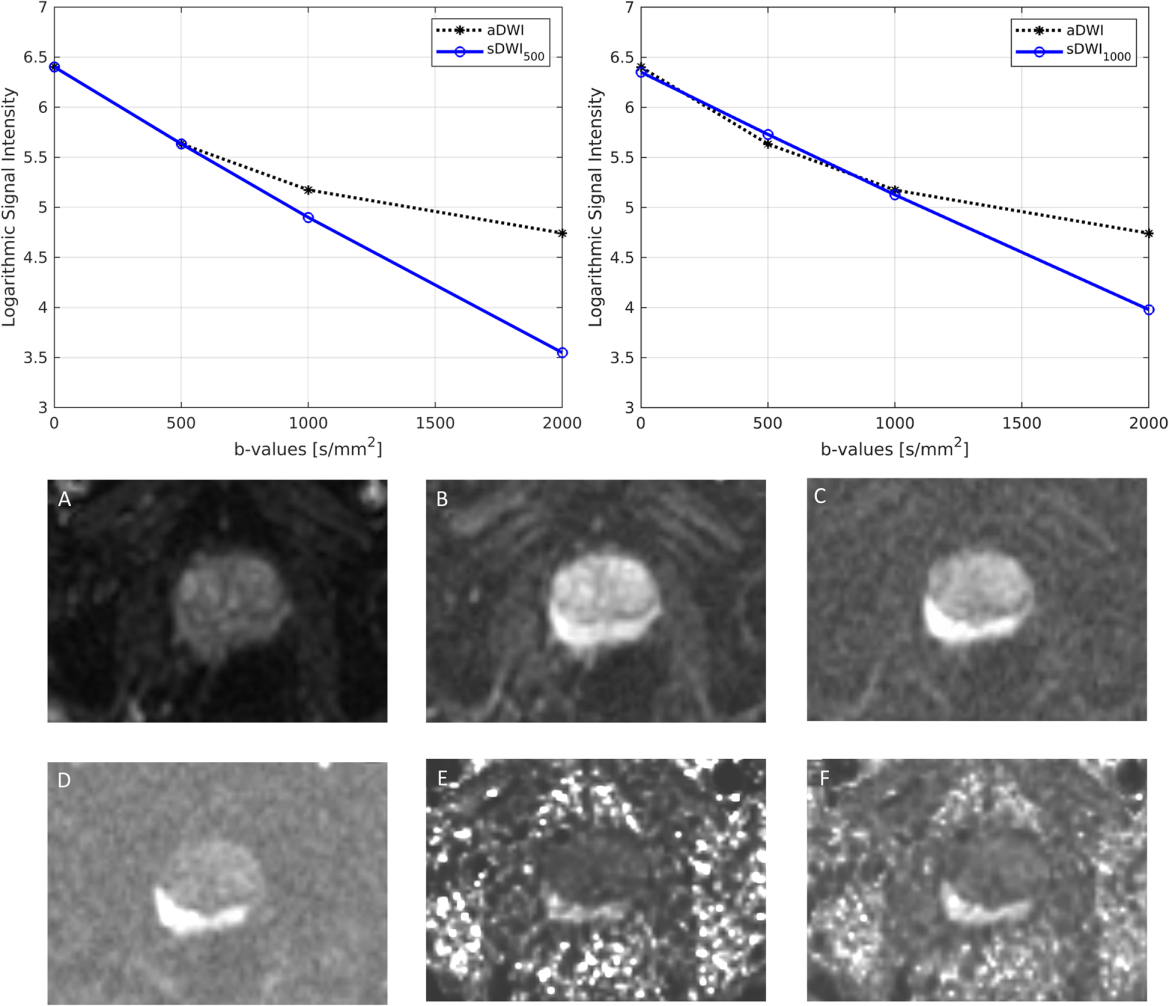
Comparison of acquired images to those synthesized with mono-exponential models are presented for one representative patient. Mean values within the prostate using either *b*-values up to 500 s/mm^2^ (sDWI_500_) or *b*-values up to 1000 (sDWI_1000_) are compared to acquired DWI (aDWI). are compared. Fig. **A**-**F** show the different diffusion images for one patient. **A**-**D** presents the acquired images for *b* = 0 s/mm^2^ (**A**), *b* = 500 s/mm^2^ (**B**), *b* = 1000 s/mm^2^ (**C**) and *b* = 2000s/mm^2^ (**D**). **E** and **F** show the synthesized *b* = 2000s/mm^2^ images. **E** shows sDWI_500_ and **F** sDWI_1000_. aDWI = acquired diffusion-weighted image for *b* = 2000s/mm.^2^

**Fig. 2:**
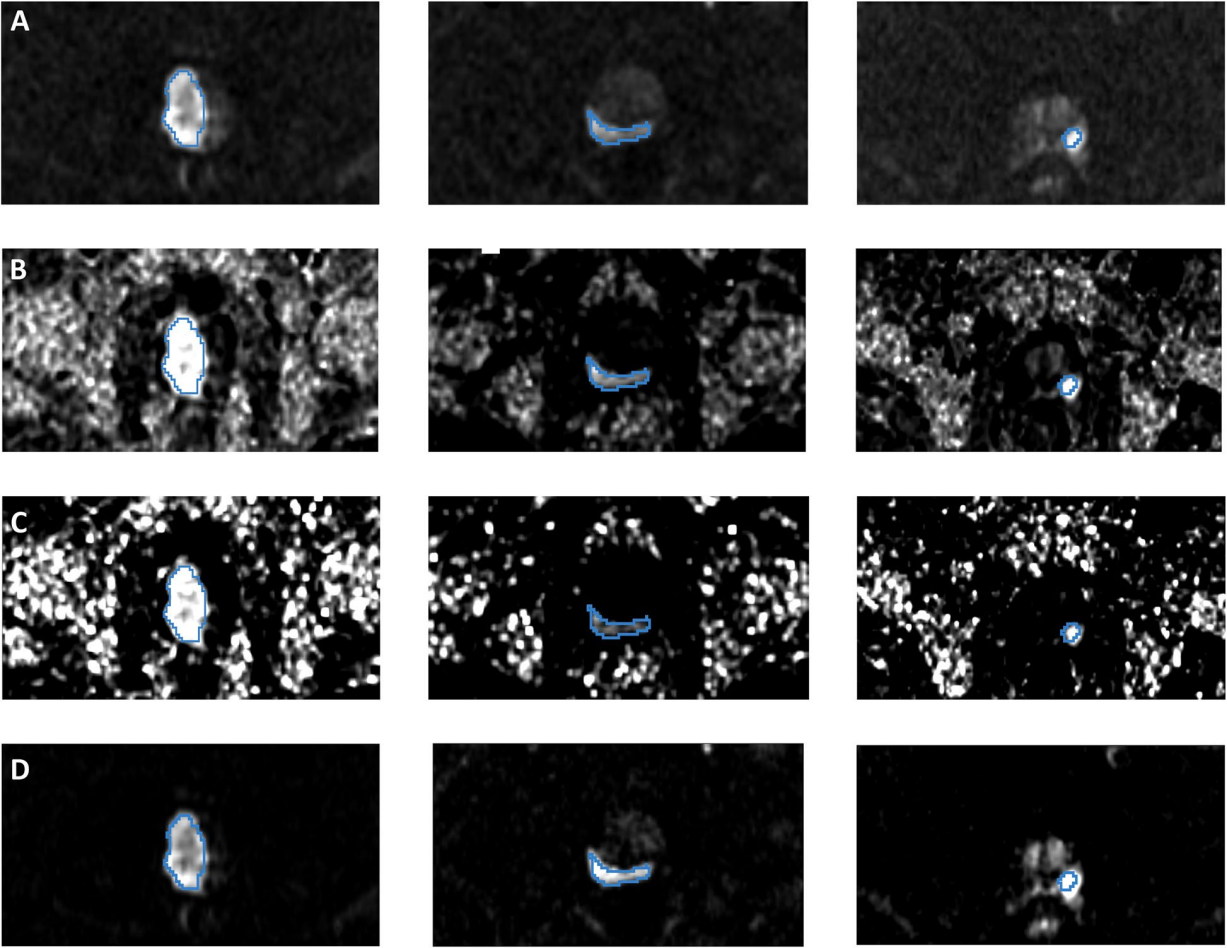
Representative images from three patients (corresponding to three columns). **A** Acquired diffusion-weighted image (aDWI) for *b* = 2000s/mm^2^, (**B**) synthesized DWI using acquired b-values up to *b* = 1000 s/mm^2^ (sDWI_1000_), (**C**) synthesized DWI using acquired values up to *b* = 500 s/mm^2^ (sDWI_500_), and (**D**) restriction spectrum imaging restriction score (RSIrs). The radiologist-defined cancer lesion for each patient is indicated in blue. All presented patients had a PI-RADS score of 5. The same window level was chosen for all presented images

Figure 1 shows the difference between acquired and synthesized *b*-values for a representative patient using the mean signal intensity within the prostate. Within the prostate, mean ± standard deviation of percent differences between sDWI and aDWI were -46 ± 35% for sDWI_1000_ and -67 ± 24% for sDWI_500_. A negative error indicates sDWI had lower intensity values than aDWI. Figure 2 shows aDWI, sDWI and RSIrs for three representative patients with the same window and level.

**Fig. 3:**
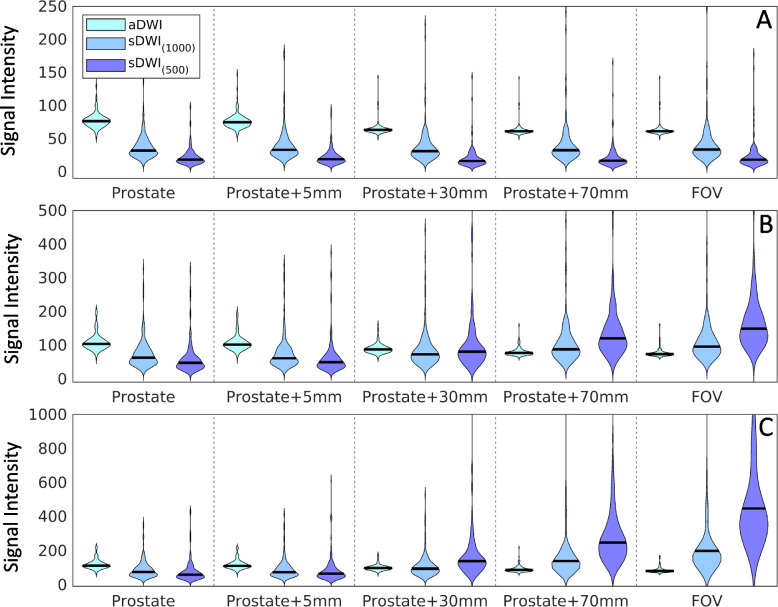
Violin plots summarizing the signal intensity across 151 patients for (**A**) 50^th^ percentile, (**B**) 95^th^ percentile, (**C**) and 98^th^ percentiles of various DWI metrics calculated for each patient. The percentiles are estimated over different regions of interest: the prostate; the prostate with varying margin (5 mm, 30 mm, or 70 mm); and the whole field of view. aDWI = acquired diffusion-weighted image with *b* = 2000s/mm^2^; sDWI = synthesized DWI for *b* = 2000s/mm^2^ using either acquired *b*-values up to 1000 s/mm^2^ (sDWI_1000_) or up to 500 s/mm.^2^ (sDWI_500_)

Comparing sDWI_1000_ to sDWI_500_, a difference of -41 ± 4% was estimated (see Table 3). sDWI_500_ had overall larger errors than sDWI_1000_. Signal intensity of sDWI was lower than aDWI in the prostate and in the prostate plus 5 mm margin considering all voxels, as indicated by a negative median difference, see Table 3. Further, a lower median value considering only the 50^th^, 95^th^ and 98^th^ percentiles was also observed. The 50^th^ percentile of aDWI is higher than sDWI for all considered ROIs. For the 95^th^ and 98^th^ percentiles, however, sDWI is larger for margins of ≥ 30 mm beyond the prostate. The standard deviation of sDWI is larger than the standard deviation of aDWI for all considered percentiles in all ROIs. A comparison of the 50^th^, 95^th^ and 98^th^ percentiles for five ROIs with varying margins around the prostate is shown in Fig. 3.

**Table 3:** Summary of median differences ((sDWI-aDWI)/aDWI) between synthesized (sDWI) and acquired (aDWI) diffusion-weighted imaging. Comparison between sDWI and aDWI is presented relative to aDWI. *FOV* Field of view

ROI	Datasets	Median Difference ± Interquartile range		
		**Total (*N* = 151)**	**csPCA (*N* = 86)**	**Benign (*N* = 65)**
Lesion	sDWI_1000_ vs. aDWI	-47 ± 36%	-43 ± 41%	-48 ± 28%
	sDWI_500_ vs. aDWI	-63 ± 27%	-60 ± 31%	-67 ± 26%
Prostate	sDWI_1000_ vs. aDWI	-55 ± 27%	-52 ± 29%	-59 ± 22%
	sDWI_500_ vs. aDWI	-72 ± 15%	-69 ± 18%	-74 ± 13%
Prostate + 5 mm margin	sDWI_1000_ vs. aDWI	-55 ± 26%	-53 ± 29%	-58 ± 21%
	sDWI_500_ vs. aDWI	-72 ± 18%	-69 ± 19%	-74 ± 13%
Prostate + 30 mm margin	sDWI_1000_ vs. aDWI	-48 ± 35%	-46 ± 41%	-50 ± 28%
	sDWI_500_ vs. aDWI	-58 ± 34%	-54 ± 45%	-61 ± 23%
Prostate + 70 mm margin	sDWI_1000_ vs. aDWI	1.9e19 ± 4.1e19%	1.8e19 ± 3.8e19%	2.0e19 ± 6.4e19%
	sDWI_500_ vs. aDWI	1.5e47 ± 1.7e48%	1.4e47 ± 1.2e48%	1.5e47 ± 3.0e48%
Whole FOV	sDWI_1000_ vs. aDWI	1.1e19 ± 2.3e19%	1.1e19 ± 2.2e19%	1.6e19 ± 2.5e19%
	sDWI_500_ vs. aDWI	3.4e48 ± 8.9e50%	2.0e48 ± 7.1e50%	1.3e49 ± 9.2e50%

Mean and standard deviation of CNR over all patients was 0.95 ± 0.87, 0.84 ± 0.80, 0.65 ± 0.66 and 0.97 ± 0.79 for aDWI, sDWI_1000,_ sDWI_500_ and RSIrs, respectively. A lower CNR indicates a lower tumor conspicuity. CNR considering only patients with csPCa changed to 1.00 ± 0.84, 0.86 ± 0.78, 0.65 ± 0.65 and 0.99 ± 0.76 for aDWI, sDWI_1000,_ sDWI_500_ and RSIrs respectively. CNR for aDWI and RSIrs proved to be significantly different to sDWI (*p* < 0.01) for all patients and patients with csPCa.

**Fig. 4:**
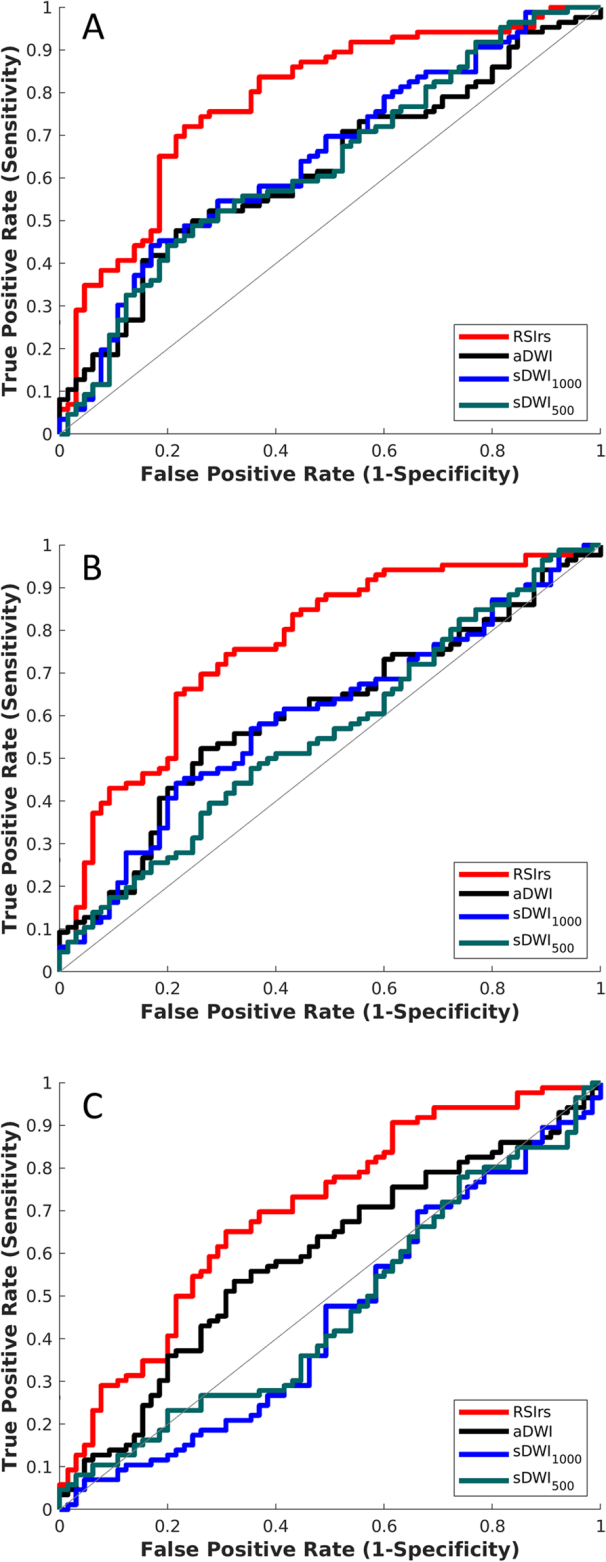
ROC curves for DWI metrics within three ROIs (**A**) the prostate, (**B**) the prostate with 5 mm margin, and (**C**) the whole field of view. The DWI metrics compared for classification accuracy are RSIrs, acquired diffusion-weighted images (aDWI), synthesized DWI using *b*-values up to 1000 s/mm^2^ (sDWI_1000_), and synthesized DWI using *b*-values up to 500 s/mm.^2^ (sDWI_500_). Clinically significant prostate cancer was defined as a Gleason score ≥ 2

**Fig. 5:**
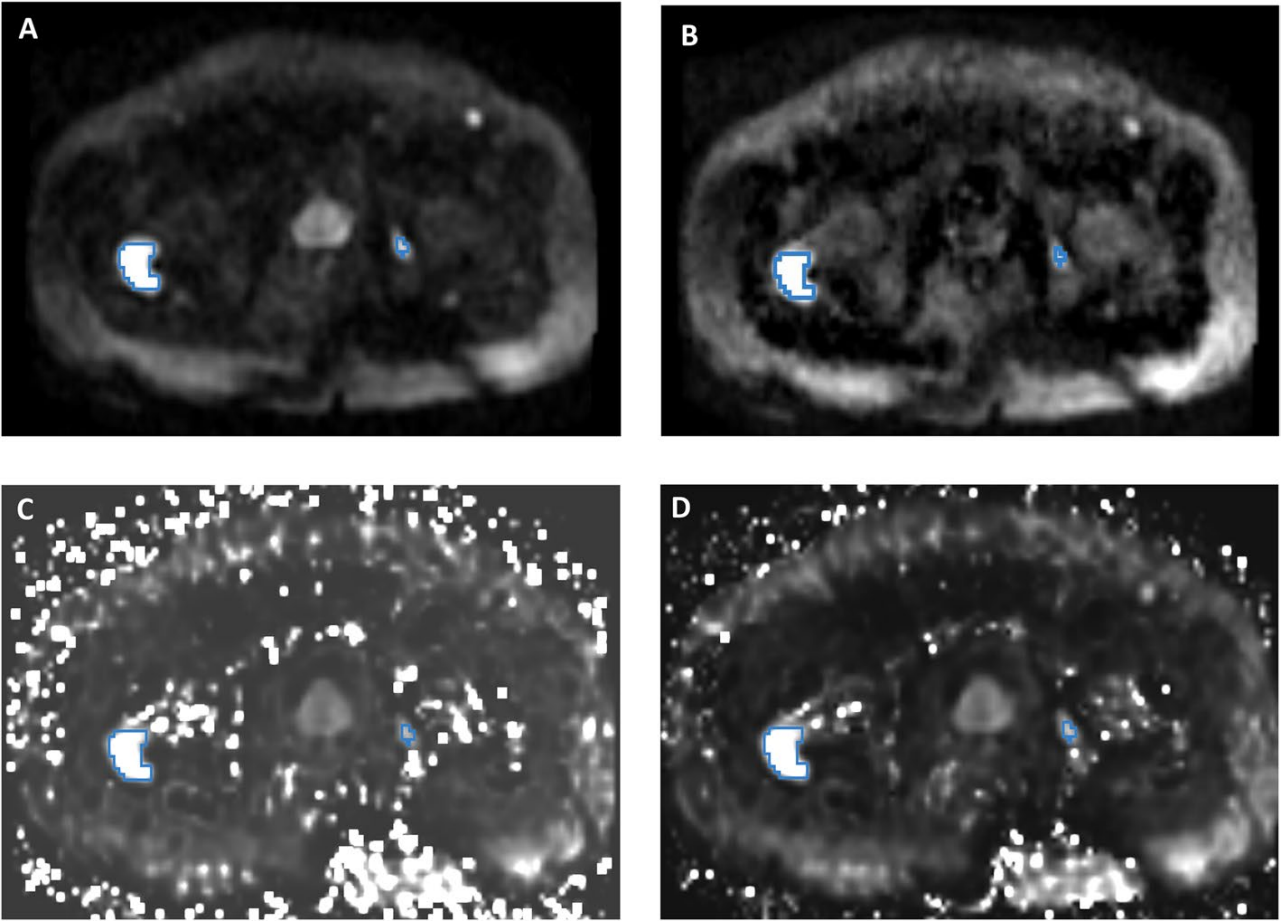
An example of pelvic DWI in a patient with prostate cancer bone metastases is shown. The blue contours mark the cancer lesions. **A** Acquired DWI with *b* = 2000 s/mm^2^; (**B**) RSIrs based on restriction spectrum imaging; (**C**) synthesized DWI using acquired *b*-values up to 500 s/mm^2^; and (**D**) synthesized DWI using acquired *b*-values up to 1000 s/mm^2^. Tumor lesions are easily detectible in **A** and **B**, but there is more high-intensity artifact on sDWI, and the smaller bone metastasis is not as easily identifiable in **C** and **D**

Figure 5 compares sDWI, aDWI and RSIrs for detection of significant cancer lesions outside of the prostate. For detection of csPCa, the AUCs for sDWI and aDWI were similar in both the prostate and prostate plus 5 mm (Fig. 4 and Table 4). Classification accuracy decreased significantly for sDWI when considering the whole FOV (AUC = 0.45 [0.36, 0.54] for sDWI_1000_ and 0.47 [0.38, 0.56] for sDWI_500_). RSIrs was superior to sDWI and aDWI for all ROIs (*p* < 0.01). The AUC of RSIrs was 0.77 [0.69, 0.84] within prostate plus 5 mm and decreased to 0.70 [0.61, 0.78] for the whole FOV. FPR90 was similar for aDWI and sDWI in all ROIs. Mean FPR90 was significantly lower for RSIrs than for either aDWI or sDWI, indicating fewer false positives (*p* < 0.05).

**Table 4:** Classification accuracy for the detection of cancer is shown for the prostate, prostate plus a 5, 30 and 70 mm margin and the whole field of view (FOV). For statistical comparison bootstrapping (*N* = 10,000) was performed and the 95% confidence intervals (CI) of AUC and the mean false positive rate at 90% sensitivity (FPR90) reported. *ROI* Region of interest, *AUC* Area under the curve, *RSIrs* Biomarker based on restriction spectrum imaging, *aDWI* Acquired diffusion *b* = 2000s/mm^2^ MRI, *sDWI*_*1000*_ Synthesized image using acquired *b*-values up to 1000 s/mm^2^, *sDWI*_*500*_ Synthesized image using acquired *b*-values up to 500 s/mm^2^, *significantly different with *p* < 0.05 in comparison to each of the other metrics

ROI	Dataset	AUC	95^th^ CI	FPR90
Prostate	RSIrs	0.78*	0.71–0.86	0.50*
	aDWI	0.62	0.53–0.71	0.82
	sDWI_1000_	0.65	0.56–0.73	0.76
	sDWI_500_	0.63	0.54–0.72	0.76
Prostate + 5 mm margin	RSIrs	0.77*	0.68–0.84	0.53*
	aDWI	0.61	0.51–0.69	0.87
	sDWI_1000_	0.60	0.51–0.69	0.84
	sDWI_500_	0.56	0.47–0.65	0.84
Prostate + 30 mm margin	RSIrs	0.72*	0.64–0.80	0.62*
	aDWI	0.58	0.49–0.67	0.89
	sDWI_1000_	0.52	0.43–0.61	0.83
	sDWI_500_	0.51	0.42–0.61	0.86
Prostate + 70 mm margin	RSIrs	0.72*	0.64–0.81	0.61*
	aDWI	0.60	0.50–0.69	0.88
	sDWI_1000_	0.46	0.36–0.55	0.87
	sDWI_500_	0.45	0.36–0.55	0.89
Whole FOV	RSIrs	0.70*	0.61–0.78	0.62*
	aDWI	0.59*	0.49–0.68	0.90
	sDWI_1000_	0.45	0.36–0.54	0.91
	sDWI_500_	0.47	0.38–0.56	0.90

## Discussion

We found that synthesized DWI images can be qualitatively similar to acquired DWI within the prostate even though sDWI is quantitatively an inaccurate representation of aDWI. Moreover, sDWI introduces unacceptable artifacts and inaccuracies in surrounding pelvic tissues. sDWI_500_ calculated using only *b*-values up to 500 s/mm^2^ was inferior to sDWI_1000_, demonstrating that the accuracy of sDWI is improved by including higher *b*-values. Acquiring additional *b*-values ≤ 1000 s/mm^2^ would further increase the accuracy of the mono-exponential fit and the calculation of sDWI, up to the theoretical limits imposed by a mono-exponential signal model. Beyond *b*-values of ~ 1000 s/mm^2^, kurtosis effects stemming from the multi-compartmental nature of tissue start to become apparent in the diffusion signal decay, which cannot be accurately modeled using a simple mono-exponential function [33]. Specifically, non-gaussian diffusion of restricted intracellular water results in more signal at high *b*-values than would be expected for purely gaussian diffusion [34], and therefore a systematic underestimation of high *b*-value signal by sDWI (Fig. 1). In this study, we showed that the median signal within the prostate was 55–72% lower on sDWI than aDWI. Ideally, *b*-values greater than 1000 s/mm^2^ should be acquired to ensure that such non-gaussian effects are appropriately measured, enabling the assessment of restricted diffusion and derived biomarkers like RSIrs. There is evidence that measurements of restricted intracellular diffusion can help to differentiate between tumors of different histological patterns, like cribriform and non-cribriform, which differ in the degree of intracellular vs extracellular water [35–37]. The clinical benefits of such granular assessment may offset the increase in scan time necessary to acquire data at higher *b*-values. Indeed, this study demonstrated a clear improvement in the detection of biopsy-proven csPCa with RSIrs compared to sDWI or aDWI.

Within the prostate and the prostate plus 5 mm margin, lesion conspicuity was reasonably preserved with sDWI. Both sDWI and aDWI had a similar quantitative performance in detecting csPCa with an AUC ranging between 0.56–0.65. However, sDWI introduced larger errors in the surrounding pelvic tissue even in a reduced FOV acquisition. Because the mono-exponential fitting was performed using a linear fit to log-transformed data for computational efficiency, areas with low SNR were susceptible to significant fitting errors. The pelvic region surrounding the prostate has many such regions with low SNR, including bone and connective tissue with inherently low SNR at the relatively long TEs used in this study, as well as fatty tissue with suppressed signal from the water-selective excitation pulse used during image acquisition.

Severe artifacts were observed on sDWI in these low-SNR regions of the pelvis surrounding the prostate, in particular for sDWI_500_, which makes the detection of metastasis outside of the prostate region difficult (Fig. 5). There are many ways to potentially improve the calculation of synthesized images including bi-exponential or multi-exponential modeling [23]. For example, RSIrs is based on a multi-exponential model and may synthesize images without introducing artifacts. Image artifacts may be explained by poor signal quality, magnitude smaller than one in a subset of voxels, or noise/distortion correction post image acquisition leading to voxels with extremely low signal intensity. In particular, mono-exponential models fail to correctly represent voxels with low signal intensity due to exponential fitting. Smoother images could be created by censoring those voxels by interpolating from surrounding voxels, smoothing low *b-*value images prior to calculation, or by thresholding low intensity voxels. For quantitative imaging, the details of such decisions would need to be clearly described and accounted for, and potentially could lead to more false positive/negative detections. Such enhanced images would not represent the measured truth and would include some unreliable voxels, which must be taken into consideration when interpreting the images. A “nicer looking” image does not necessarily mean that the image quality or reliability is better.

Prior studies reported sDWI to have higher subjective quality and tumor conspicuity [5, 27]. This may reflect the particular imaging sequences and, platforms used, or the particular image enhancement effects. It is also important to note that these prior results were mostly subjective judgements and not quantitative assessments of imaging quality. In the presented study we have proven that tumor conspicuity is quantitatively greater with aDWI (CNR = 0.95) in comparison to sDWI (CNR = 0.65–0.84). However, an in-depth reader study would be necessary to evaluate the benefits of sDWI over aDWI for contouring, and it might be an interesting topic for future studies. The accuracy in detecting csPCa on a patient-level was assessed by ROC curves. The AUCs proved to be similar for sDWI and aDWI in both the prostate and prostate plus 5 mm. An option to improve the accuracy of DWI is to use a multi-compartment DWI model, e.g., RSIrs, for a more stable and accurate signal extrapolation. For example, RSIrs outperformed both sDWI and aDWI in the present study, see Fig. 4. Other promising multi-compartment models have proved to be superior to conventional multiparametric MRI, like a biomarker derived from VERDICT that outperformed ADC in the detection of csPCa [14]. Hybrid multidimensional MRI acquisitions also showed promising results for classifying csPCa with a reported AUC of 0.94 [33].

One limiting factor of our study was that we only considered a retrospective dataset from a single scanner and a single institution. Also, the slice thickness of 6 mm for DWI data analyzed here is larger than the 4 mm recommended in PI-RADS v2.1. The larger through-plane voxel size increases signal-to-noise ratio but it is possible some very small tumors could have gone undetected, though any such inaccuracies would apply to aDWI, sDWI, and RSIrs alike. Further, only the conventional mono-exponential model was tested in the presented study, as this is the conventional method most cited and used for synthesis of high *b-*value DWI. A precise comparison of all possible methods for synthesizing DWI is beyond the scope of this manuscript. RSIrs is one quantitative biomarker based on a multi-compartment model. The acquisition protocol in the datasets here was not optimized for models like hybrid multi-dimensional MRI or VERDICT.

## Conclusions

Within the prostate, sDWI is a systematically inaccurate representation of aDWI, but the techniques are quantitatively comparable in terms of detecting csPCa with an AUC range between 0.56–0.65. In the surrounding pelvic tissue, high signal intensity artifacts are introduced with sDWI. These artifacts decrease CNR and thus affect the csPCa detection sensitivity in surrounding tissues and might mask potential metastases within the pelvis. RSIrs is superior to either sDWI or aDWI for quantitative csPCa detection. Despite the quantitative inaccuracies, sDWI may still be adequate for current subject clinical interpretation within the prostate.

## Data Availability

The datasets generated during and/or analyzed during the current study are not publicly available due to required IRB approval but are available from the corresponding author on reasonable request.

## Abbreviations

DWI: Diffusion-weighted imaging
csPCa: Clinically significant prostate cancer
FOV: Field of view
RSIrs: Restriction spectrum imaging restriction score
CNR: Contrast-to-noise ratio
sDWI: Synthesized diffusion-weighted image
aDWI: Acquired diffusion-weighted image
ROI: Region of interest
ROC: Receiver operating characteristic
AUC: Area under the curve
FPR90: False positive rate at 90% sensitivity
SNR: Signal-to-noise ratio
